# Intensive Rehabilitation With Adjunctive Bilateral Anodal tDCS in Post‐Stroke Dysphagia: A Multicenter Randomized Controlled Trial

**DOI:** 10.1111/ene.70686

**Published:** 2026-07-01

**Authors:** Giuseppe Cosentino, Tommaso Bocci, Francesca Cecchi, Valeria Pingue, Carla Giudice, Nicole Pizzorni, Antonello Grippo, Simone Pierro, Ilaria Pellegrini, Emanuela Concas, Sara Rocca, Simone Mauramati, Giulia Bertino, Valentina Grillo, Shaheen Hamdy, Alberto Priori, Antonio Schindler, Antonio Nardone, Cristina Tassorelli, Enrico Alfonsi

**Affiliations:** ^1^ Department of Brain and Behavioral Sciences University of Pavia Pavia Italy; ^2^ IRCCS Mondino Foundation Pavia Italy; ^3^ Department of Health Sciences, “Aldo Ravelli” Center for Neurotechnology and Experimental Brain Therapeutics University of Milan Milan Italy; ^4^ Clinical Neurology Unit, Department of Health Sciences, “Azienda Socio‐Sanitaria Territoriale Santi Paolo e Carlo” University of Milan Milan Italy; ^5^ Dipartimento di Medicina Sperimentale e Clinica Università degli Studi di Firenze Florence Italy; ^6^ IRCCS Fondazione don Carlo Gnocchi Firenze Italy; ^7^ Department of Clinical‐Surgical, Diagnostic and Pediatric Sciences University of Pavia Pavia Italy; ^8^ Neurorehabilitation and Spinal Units of Pavia Institute Istituti Clinici Scientifici Maugeri IRCCS Pavia Italy; ^9^ Department of Biomedical and Clinical Sciences Università degli Studi di Milano Milan Italy; ^10^ SODC Neurofisiopatologia AOUC Azienda Ospedaliero‐Universitaria Careggi Firenze Italy; ^11^ Department of Otolaryngology University of Pavia, IRCCS Policlinico San Matteo Foundation Pavia Italy; ^12^ Division of Diabetes, Endocrinology and Gastroenterology, School of Medical Sciences, Centre for Gastrointestinal Sciences, Faculty of Biology, Medicine and Health, Salford Royal Foundation Trust University of Manchester Manchester UK; ^13^ Headache Science and Neurorehabilitation Center IRCCS Mondino Foundation Pavia Italy

**Keywords:** dysphagia, neurostimulation, speech‐language therapy, stroke, transcranial direct current stimulation

## Abstract

**Background:**

Oropharyngeal dysphagia is a common and disabling consequence of stroke. Transcranial direct current stimulation (tDCS) has shown potential in promoting swallowing recovery, although evidence remains limited.

**Objective:**

To determine whether bilateral anodal tDCS combined with intensive speech‐language therapy (SLT) improves swallowing outcomes compared with sham stimulation in patients with post‐stroke dysphagia. Exploratory analyses examined the influence of treatment phase, sex, lesion site, and baseline severity.

**Methods:**

This multicenter, randomized, double‐blind, sham‐controlled trial enrolled patients with supratentorial or infratentorial ischemic stroke and oropharyngeal dysphagia. Participants received either bilateral anodal tDCS or sham stimulation (1.5 mA, 20 min/day, 5 days/week for 2 weeks) combined with intensive SLT over 6 weeks. Swallowing outcomes were assessed at baseline, 2 weeks, and 6 weeks using the Dysphagia Outcome and Severity Scale (DOSS, primary outcome), Penetration–Aspiration Scale (PAS), Mann Assessment of Swallowing Ability (MASA), and Swallowing Quality of Life questionnaire (SWAL‐QoL).

**Results:**

Forty‐six patients (24 active, 22 sham) completed the protocol. Both groups showed significant improvement across all outcomes (*p* < 0.001), with no significant difference between active and sham stimulation. The DOSS was the most sensitive measure, showing sustained improvement over time. Exploratory analyses indicated greater MASA gains with active tDCS in infratentorial strokes (*p* = 0.04). Correlation analyses showed that greater baseline dysphagia severity was associated with larger functional gains.

**Conclusions:**

Intensive SLT was associated with meaningful recovery in post‐stroke dysphagia, regardless of stimulation condition. Exploratory findings suggest that bilateral tDCS may confer additional benefit in selected lesion subgroups.

## Graphical abstract

In this multicenter randomized controlled trial, intensive speech‐language therapy was associated with significant improvement in swallowing outcomes in patients with post‐stroke dysphagia. No significant differences between active bilateral anodal transcranial direct current stimulation and sham stimulation were observed in the overall cohort. Exploratory analyses suggested greater improvement on the Mann Assessment of Swallowing Ability in patients with infratentorial stroke receiving active stimulation, supporting a possible subgroup‐specific benefit of bilateral tDCS.

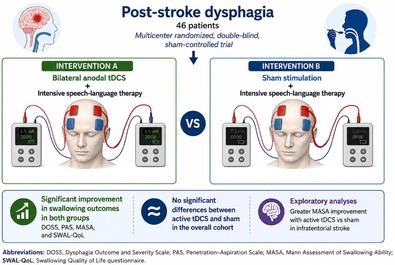

## 1 Introduction

Oropharyngeal dysphagia is among the most frequent and disabling sequelae of stroke, with an estimated incidence ranging from 20% to 81% depending on lesion site, timing, and diagnostic method [1]. Dysphagia is not only a marker of poor functional outcome but also a major determinant of morbidity and mortality, leading to aspiration pneumonia, malnutrition, dehydration, prolonged hospitalization, and reduced quality of life [2, 3]. Spontaneous recovery may occur, particularly in the subacute phase, yet a substantial proportion of patients exhibit persistent dysfunction, often requiring long‐term enteral feeding or compensatory strategies [4]. Standard speech and language therapy (SLT) remains the cornerstone of management, but its efficacy as a stand‐alone treatment is limited, prompting the exploration of adjunctive approaches.

Over the last two decades, non‐invasive brain stimulation techniques have emerged as promising tools to modulate cortical excitability and plasticity. Among these, transcranial direct current stimulation (tDCS) has gained particular interest for its ability to induce polarity‐dependent changes in neuronal membrane potentials, with anodal stimulation increasing cortical excitability and cathodal stimulation decreasing it [5, 6, 7]. Experimental studies demonstrated that anodal tDCS over the swallowing motor cortex can enhance cortico‐pharyngeal excitability and promote functional changes in bilateral swallowing networks [8, 9, 10], providing the rationale for clinical trials.

Several randomized controlled trials (RCTs) have explored the efficacy of tDCS in post‐stroke dysphagia with encouraging but heterogeneous results [11, 12, 13]. Unilateral anodal stimulation over the intact hemisphere improved swallowing function in acute patients [14], whereas bilateral protocols in chronic cases failed to show superiority over sham despite within‐group gains [15]. Contralesional anodal tDCS proved effective in acute stroke and was associated with enhanced cortical activation [16], while a recent comparison of ipsilesional, contralesional, and bilateral montages identified bilateral stimulation as the most beneficial approach [17]. Infratentorial strokes, however, have been scarcely investigated, with only limited evidence supporting contralesional anodal stimulation in acute cases [18].

Overall, despite promising findings, major uncertainties persist regarding the optimal montage, the most responsive recovery phase, and the potential interaction between neuromodulation and intensive SLT.

In the present randomized, double‐blind, multicenter trial, we evaluated the clinical effects of bilateral anodal tDCS applied over the primary sensorimotor swallowing cortices, combined with intensive SLT, in patients with supratentorial or infratentorial ischemic stroke. Recruitment covered a broad but clinically relevant window from 72 h to 6 months, excluding hyperacute and chronically stabilized cases.

The primary aim was to assess whether bilateral anodal tDCS improves swallowing outcomes on the Dysphagia Outcome and Severity Scale (DOSS) compared with sham. Secondary endpoints included the Penetration–Aspiration Scale (PAS), the Mann Assessment of Swallowing Ability (MASA), and the Swallowing Quality of Life questionnaire (SWAL‐QOL). The trial also explored whether lesion location (supratentorial vs. infratentorial) and treatment timing (early vs. late) influence responsiveness, with the goal of clarifying the role of bilateral tDCS as an adjunct to intensive rehabilitation and informing patient selection and optimal timing for neuromodulatory interventions in post‐stroke dysphagia.

## 2 Materials and Methods

### 2.1 Study Population

Consecutive patients with post‐stroke oropharyngeal dysphagia were screened between June 2021 and May 2025 across five Italian centers. Recruitment was partly limited by organizational constraints related to the COVID‐19 pandemic during a significant portion of the study period. In addition, the requirement for FEES‐confirmed dysphagia and participation in a standardized 6‐week intensive rehabilitation program reduced eligibility and feasibility, particularly in post‐acute patients, in whom continuation of treatment during hospitalization was not consistently feasible across participating centers depending on local admission criteria and length of stay. Eligible participants were adults aged 18 years or older with a first‐ever or recurrent ischemic stroke confirmed by neuroimaging, and evidence of oropharyngeal dysphagia defined by a DOSS score ≤ 6 on fiberoptic endoscopic evaluation of swallowing (FEES), indicating at least mild functional impairment in swallowing. Although some patients with a DOSS score of 6 may exhibit functionally safe swallowing, their inclusion was deemed appropriate to account for possible deterioration and real‐world clinical variability. Patients were enrolled if the time from stroke onset ranged between 72 h and 6 months, provided they were able to participate in SLT and gave written informed consent.

For exploratory purposes, patients were also classified according to the timing of treatment initiation into an early phase (therapy started between 72 h and 4 weeks post‐stroke, thus excluding hyperacute cases) and a late phase (therapy started between 5 weeks and 6 months post‐stroke, excluding chronically stabilized patients). This temporal categorization was based on evidence that post‐stroke neuroplasticity peaks within the first few weeks and progressively declines thereafter, marking the transition from subacute to early chronic recovery phases [12, 19, 20].

Exclusion criteria included pre‐existing dysphagia unrelated to stroke, severe cognitive impairment interfering with therapy participation, progressive neurological or oncological diseases, and standard contraindications to tDCS such as metallic cranial implants, pacemakers, or other implanted electronic devices, history of seizures or active epilepsy, and dermatological conditions at the electrode sites.

### 2.2 Study Design

This was a randomized, double‐blind, sham‐controlled, multicenter clinical trial. Patients were allocated 1:1 to bilateral anodal tDCS or sham stimulation using centralized randomization. Both patients and outcome assessors were blinded to group allocation. All participants received standardized intensive SLT. Assessments were conducted at baseline, after 2 weeks (end of stimulation), and after 6 weeks (end of SLT).

The trial was registered retrospectively at ClinicalTrials.gov (Identifier: NCT07152899). Delayed registration was due to administrative constraints related to the COVID‐19 pandemic. The study protocol, primary outcome, and statistical analysis plan were defined prior to data analysis and were not modified after study initiation.

### 2.3 Intervention Protocol

#### 2.3.1 Transcranial Direct Current Stimulation (tDCS)

Transcranial direct current stimulation (tDCS) was delivered using CE‐certified stimulators, which differed across participating centers but all adhered to the same stimulation protocol and international safety standards. For active treatment, two anodal electrodes (5 × 7 cm) were placed bilaterally over the orofacial swallowing cortices, centered 3.5 cm lateral and 1 cm anterior to the vertex, with the long axis parallel to the central sulcus. This montage was designed to target the primary sensorimotor representation of swallowing [9, 16]. Each cathodal electrode (10 × 10 cm) was placed supraorbitally, contralateral to its anode. Stimulation intensity was 1.5 mA for 20 min per session, once daily, 5 days per week, for 2 weeks (10 sessions total). Sham stimulation used the same electrode placement, but current was delivered only for 30 s at the beginning and again for 30 s at the end of each session to mimic transient cutaneous sensations without producing sustained cortical effects.

#### 2.3.2 Speech and Language Therapy (SLT)

All participants underwent intensive SLT delivered by experienced therapists, consisting of 40‐min daily sessions, 5 days per week, for six consecutive weeks. During the first 2 weeks, SLT was performed concurrently with active or sham transcranial direct current stimulation (tDCS) and continued afterward to exploit potential synergistic effects between neuromodulation and task‐specific training. From week three onward, patients continued with SLT alone following the same schedule. The program included individualized combinations of swallowing and non‐swallowing exercises, compensatory and postural strategies, and progressive oropharyngeal strengthening tasks, tailored to each patient's swallowing profile. The structure and content of the rehabilitation protocol are summarized in Table S1.

#### 2.3.3 Outcome Measures

**FIGURE 1:**
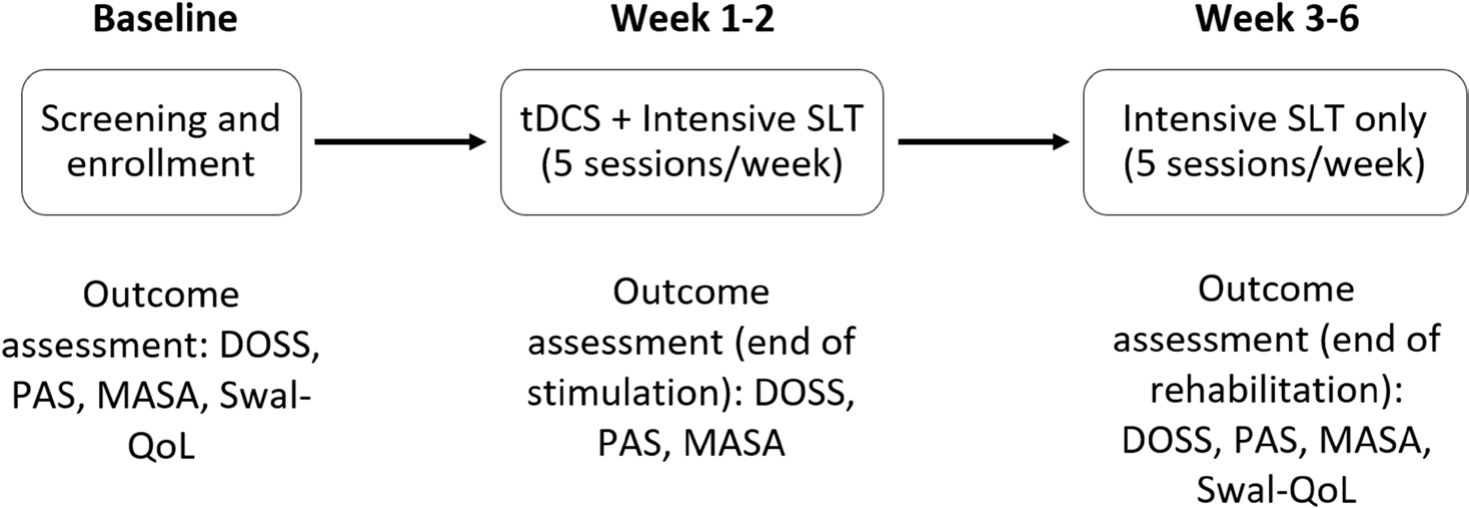
Study timeline. Temporal sequence of bilateral anodal transcranial direct current stimulation (tDCS) and intensive speech‐language therapy (SLT) interventions, together with the timing of outcome assessments. DOSS, Dysphagia Outcome and Severity Scale; MASA, Mann Assessment of Swallowing Ability; PAS, Penetration–Aspiration Scale; SWAL‐QoL, Swallowing Quality of Life questionnaire.

The primary outcome was improvement in swallowing function as assessed by the Dysphagia Outcome and Severity Scale (DOSS) [21]. Secondary outcomes comprised the Penetration–Aspiration Scale (PAS) [22], which evaluates airway safety during swallowing, and the Italian version of the Mann Assessment of Swallowing Ability (MASA) [23], a standardized clinical tool for the global assessment of swallowing performance. DOSS and PAS scores were assigned based on instrumental swallowing assessments performed at each time point using FEES. The overall study timeline, including the sequence of interventions and assessment time points, is shown in Figure 1. During each assessment, all patients were tested with thin liquids (IDDSI level 0, blue‐dyed water; 5, 10, and 15 mL) and pureed consistency (IDDSI level 4, blue‐dyed fruit gel/pureed apple; 5, 10, and 15 mL), according to the International Dysphagia Diet Standardization Initiative (IDDSI) framework. In patients able to tolerate a solid bolus, solid consistency (IDDSI level 7, half cracker) was additionally tested. Three trials were administered for each volume and consistency. The protocol could be reduced at the individual level according to swallowing safety. For both scales, scoring was based on the worst performance across tested consistencies. All evaluations were performed by trained clinicians blinded to treatment allocation. In addition, patient‐reported outcomes were collected through the Italian version of the Swallowing Quality of Life questionnaire (SWAL‐QOL) [24], to assess the perceived impact of dysphagia and its treatment on everyday life.

#### 2.3.4 Sample Size Calculation and Statistical Analysis

Power analysis was performed in G*Power 3.0.10 (Heinrich Heine University, Düsseldorf, Germany) using a Wilcoxon signed‐rank test for paired samples (two‐sided *α* = 0.05). Based on estimates from Ahn et al. [15] on the DOSS (pre: mean = 3.46, SD = 1.27; post: mean = 4.08, SD = 1.50), a sample size of 38 patients was required to detect the within‐group pre–post difference with 90% power, corresponding to 19 per arm if evenly distributed. Accordingly, the study was primarily powered to detect within‐group changes over time rather than modest between‐group differences between active and sham stimulation. To account for attrition and preserve statistical power, the target enrollment was increased to 48 patients (24 per group).

As outcome measures did not show a normal distribution according to the Shapiro–Wilk test, non‐parametric tests were applied. Within‐group changes over time were analyzed using the Friedman test for repeated measures, the non‐parametric equivalent of a one‐way repeated measures ANOVA. When significant effects were found, Wilcoxon signed‐rank post hoc tests with Bonferroni correction were performed (adjusted significance threshold *p* < 0.0167). Treatment effects were further evaluated by calculating delta scores (change from baseline to 2 and 6 weeks), which were compared between groups using Mann–Whitney *U* tests.

Subgroup analyses explored the potential influence of lesion location (supratentorial vs. infratentorial), treatment timing (early ≤ 4 weeks vs. late > 4 weeks), and sex. Correlations between clinical improvement and baseline variables (age, NIHSS, time since stroke) were assessed using Spearman's rho.

For all analyses, *p* < 0.05 was considered as significant. The statistical analyses were performed using Statsoft's Statistica version 14.2.0.

#### 2.3.5 Ethical Approval

This study was conducted in accordance with the Declaration of Helsinki and was approved by the Ethics Committee of Fondazione IRCCS Policlinico San Matteo, Pavia, Italy (protocol 20210006510; January 22, 2021). Written informed consent was obtained from all participants prior to inclusion.

Artificial intelligence tools (ChatGPT, OpenAI) were used solely for language editing. All scientific content and interpretations were verified by the authors.

## 3 Results

### 3.1 Study Population

**FIGURE 2:**
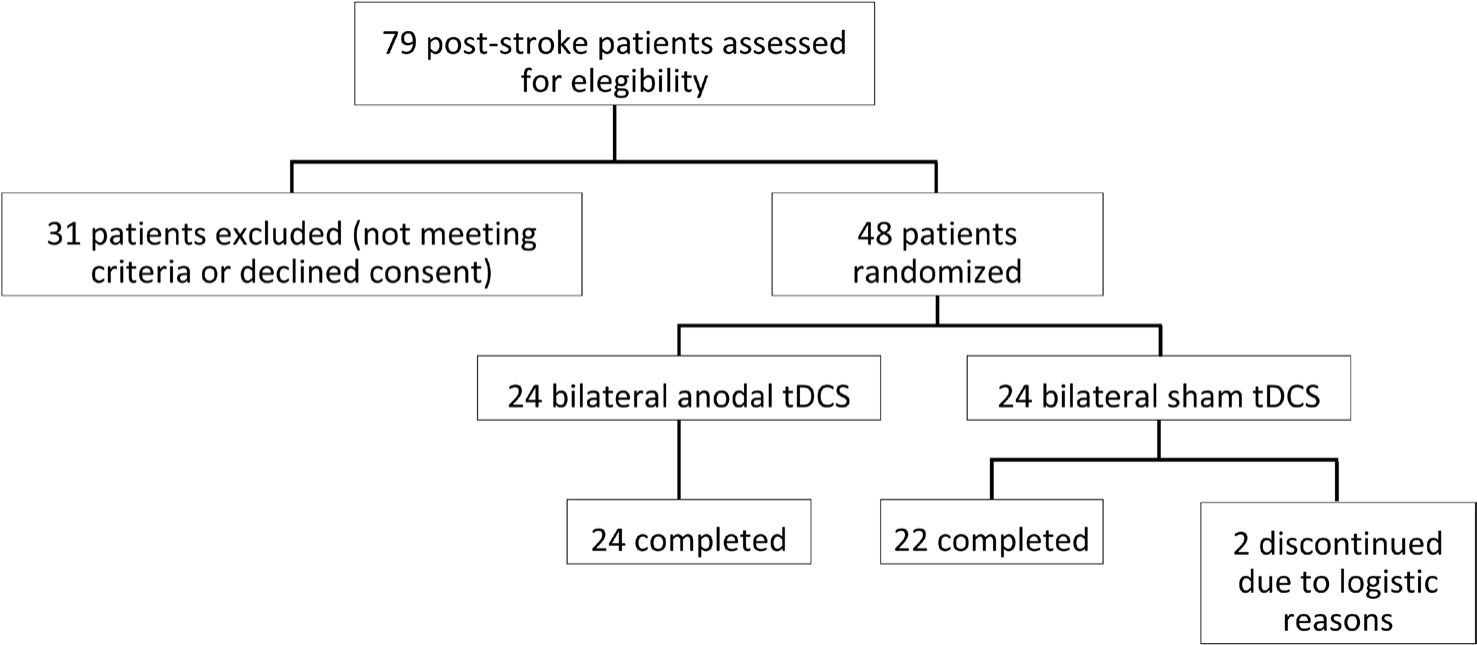
Participant flow. CONSORT diagram depicting the recruitment, randomization, and analysis of patients with post‐stroke oropharyngeal dysphagia across five Italian centers.

Out of 79 stroke patients assessed for eligibility, 31 did not meet inclusion criteria or declined participation. A total of 48 patients fulfilled eligibility criteria and were randomized: 24 to bilateral anodal tDCS and 24 to sham stimulation. Two participants in the sham group discontinued treatment for logistic reasons, resulting in 46 patients completing the intervention (24 active, 22 sham) (Figure 2).

The study cohort comprised 14 females and 32 males (Table 2). Sex distribution did not differ significantly between supratentorial and infratentorial strokes. Mean age and time since onset were comparable across lesion locations. Within the supratentorial subgroup, 11 patients had left‐hemispheric lesions and 15 had right‐hemispheric lesions. Due to the limited sample size, lesion laterality was not included in formal subgroup analyses.

Baseline clinical and demographic characteristics did not differ significantly between the active and sham tDCS groups.

At baseline, patients with infratentorial strokes showed more severe dysphagia than those with supratentorial lesions, as reflected by lower DOSS and higher PAS scores, with a trend toward lower MASA values. Baseline severity also differed by sex, with males presenting greater impairment than females. These differences are summarized in Table 1.

**TABLE 1:** Demographic and baseline clinical characteristics of the study population, stratified by lesion location (infratentorial vs. supratentorial), sex (female vs. male), and stimulation group (active tDCS vs. sham).

	All patients	Infratentorial	Supratentorial	*p* (Infra vs. Supra)	Male	Female	*p* (F vs. M)	Sham tDCS	Active tDCS	*p* (Sham vs. Active)
Age (years)	65.2 (13.4)	63.1 (12.4)	66.8 (14.2)	0.36	63.3 (12.8)	69.5 (14.3)	0.15	67.9 (12.6)	62.7 (14)	0.19
Sex	14F/32M	3F/17M	11F/15M	0.09	—	—	—	6F/16M	8F/16M	0.58
Days from onset	57.3 (77.8)	53.7 (71.6)	60.0 (83.5)	0.79	69.7 (89.8)	28.8 (21)	0.1	60.9 (50.2)	37.2 (44.8)	0.10
NIHSS	9 (5)	6.8 (4.7)	10.6 (4.7)	**0.01**	8.3 (4.9)	10.5 (5.1)	0.2	9.7 (5.5)	8.3 (4.6)	0.37
DOSS	3 (1.6)	2 (1.2)	3.7 (1.5)	**0.00001**	2.5 (1.5)	4.1 (1.5)	**0.001**	3.1 (1.5)	2.9 (1.8)	0.75
PAS	3.6 (2.3)	5.1 (2.4)	2.4 (1.4)	**0.00002**	4.2 (2.4)	2.1 (1.3)	**0.005**	3.8 (2.4)	3.4 (2.3)	0.54
MASA	156 (25.7)	147.7 (26.6)	162.2 (23.5)	0.06	153.2 (27.1)	162.0 (21.9)	0.3	154.1 (28.1)	157.6 (23.7)	0.16
Swal‐QoL	144.1 (22.3)	143.7 (26.5)	164.5 (18.5)	0.92	143.7 (23.4)	146.4 (17.8)	0.8	141.4 (22.8)	147.5 (22.1)	0.74

*Note:* Values are expressed as mean ± standard deviation (SD). Between‐group comparisons were performed using the independent‐samples *t*‐test or the Mann–Whitney *U* test for continuous variables according to data distribution, as determined by the Shapiro–Wilk test. The Chi‐square test was used for categorical variables. Bold *p* values indicate statistically significant differences (*p* < 0.05).

Abbreviations: DOSS, Dysphagia Outcome and Severity Scale; MASA, Mann Assessment of Swallowing Ability; NIHSS, National Institutes of Health Stroke Scale; PAS, Penetration–Aspiration Scale; SWAL‐QoL, Swallowing Quality of Life questionnaire.

### 3.2 Clinical Outcomes

The stimulation procedure was well tolerated, and no adverse events or side effects were reported in any patient. Participants in both groups reported comparable sensory experiences during stimulation, most commonly transient tingling or current sensation at the beginning and at the end of stimulation. No participant explicitly indicated being able to reliably distinguish active from sham treatment. Although formal quantitative assessment of blinding success was not performed, these qualitative observations suggested that blinding was maintained throughout the study. Changes in swallowing outcomes over time are summarized in Table 2. Overall, both treatment groups showed significant improvement across all outcome measures throughout the study period.

**TABLE 2:** Clinical changes from baseline in swallowing outcome measures at 2‐week and 6‐weeks in the active bilateral anodal tDCS and sham groups.

	Baseline	2 week follow‐up	6 week follow‐up	Friedman test significativity	Significant differences at post hoc analyses (Bonferroni correction)
DOSS					
Anodal tDCS	2.9 ± 1.8	4.0* ± 2.0	4.7*, ** ± 2.1	*p* = 0.000001	Bas vs. 2 weeks *p* = 0.0005; Bas vs. 6 weeks *p* = 0.00003; follow‐up 2 weeks vs. 6 weeks *p* = 0.005
Sham tDCS	3.1 ± 1.5	3.9* ± 1.9	4.4*, ** ± 2.0	*p* = 0.0001	Bas vs. 2 weeks *p* = 0.007; Bas vs. 6 weeks *p* = 0.00007; follow‐up 2 weeks vs. 6 weeks *p* = 0.005
PAS					
Anodal tDCS	3.4 ± 2.3	2.8 ± 2.5	2.6** ± 2.5	*p* = 0.0006	Bas vs. 6 weeks *p* = 0.01
Sham tDCS	3.8 ± 2.4	3.3 ± 2.3	2.9** ± 2.3	*p* = 0.003	Bas vs. 6 weeks *p* = 0.01
MASA					
Anodal tDCS	158 ± 23.7	171* ± 21.9	176*, ** ± 22.5	*p* = 000001	Bas vs. 2 weeks *p* = 0.000008; Bas vs. 6 weeks *p* = 0.000005; follow‐up 2 weeks vs. 6 weeks *p* = 0.01
Sham tDCS	154* ± 28.1	164* ± 30.6	169*, ** ± 31.6	*p* = 0.00001	Bas vs. 2 weeks *p* = 0.004; Bas vs. 6 weeks *p* = 0.0002; follow‐up 2 weeks vs. 6 weeks *p* = 0.001
Swal‐QoL					
Anodal tDCS	142 ± 15.6	—	176 ± 18.6	*p* = 0.02	—
Sham tDCS	148 ± 16.8	—	183.2 ± 13.5	*p* = 0.0006	—

*Note:* Values are expressed as mean ± standard deviation (SD). Within‐group effects were analyzed using the Friedman test for repeated measures followed by Wilcoxon signed‐rank post hoc tests with Bonferroni correction (*p* < 0.0167). Between‐group comparisons (active vs. sham) were conducted on delta values (2 weeks—baseline; 6 weeks—baseline) and are presented in Figures 3 and 4.

Abbreviations: Bas, Baseline; DOSS, Dysphagia Outcome and Severity Scale; MASA, Mann Assessment of Swallowing Ability; PAS, Penetration–Aspiration Scale; SWAL‐QoL, Swallowing Quality of Life questionnaire; tDCS, transcranial direct current stimulation.

* Significant difference vs. baseline (*p* < 0.05 after Bonferroni correction).

** Significant difference between 6‐week and 2‐week follow‐up (*p* < 0.05 after Bonferroni correction).

**FIGURE 3:**
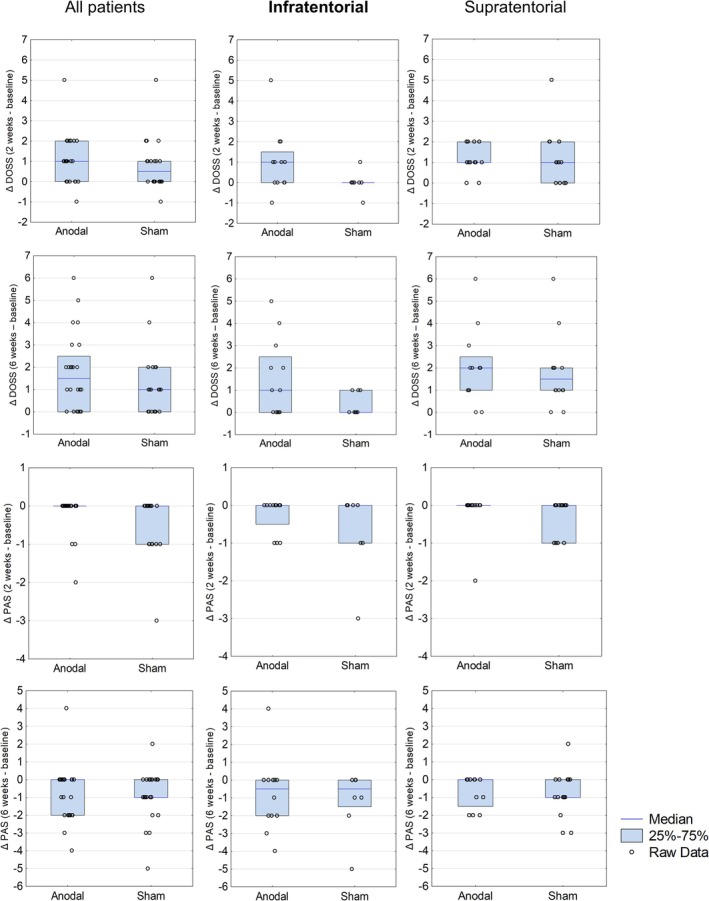
Changes in swallowing outcomes. Delta (Δ) values for the Dysphagia Outcome and Severity Scale (DOSS) and the Penetration–Aspiration Scale (PAS) at 2 and 6 weeks in the bilateral anodal tDCS and sham groups. Individual data points are shown for the overall cohort as well as for infratentorial and supratentorial subgroups. Positive Δ values indicate improvement in swallowing function (DOSS), whereas negative Δ values indicate reduced airway penetration and aspiration events, reflecting improved swallowing safety (PAS).

For the DOSS, which represented the primary endpoint, patients receiving bilateral anodal tDCS exhibited a steady increase in scores from baseline to 2 weeks (end of stimulation) and further improvement at 6 weeks (end of intensive SLT). A similar pattern was observed in the sham group, indicating functional recovery under intensive rehabilitation. Comparisons of delta values (i.e., 2 weeks—baseline and 6 weeks—baseline) showed no significant differences between the two groups (Figure 3).

**FIGURE 4:**
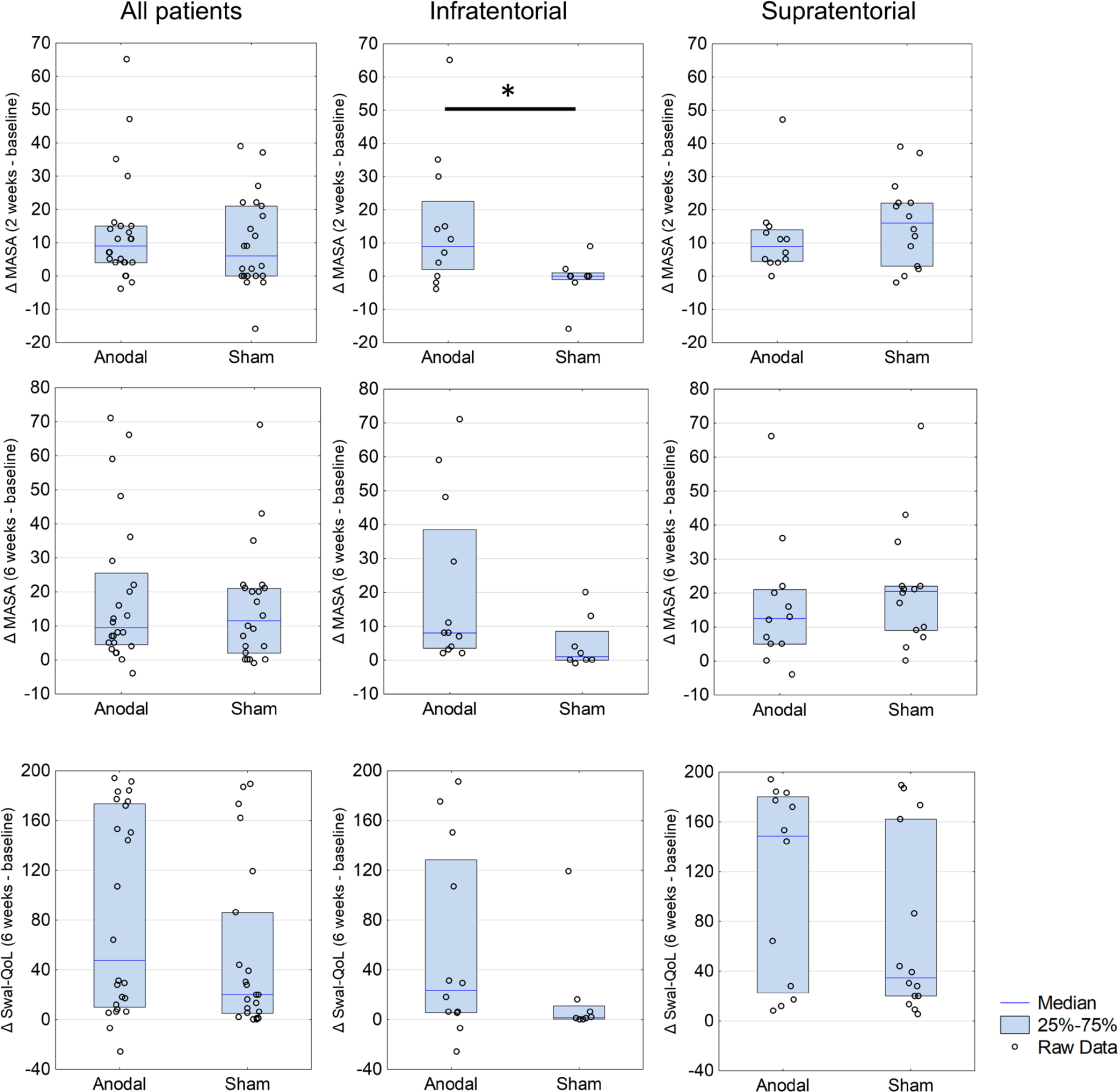
Changes in swallowing ability and quality of life. Delta (Δ) values for the Mann Assessment of Swallowing Ability (MASA) at 2 and 6 weeks and for the Swallowing Quality of Life questionnaire (SWAL‐QoL) at 6 weeks in the bilateral anodal tDCS and sham groups. Individual data points are shown for the overall cohort as well as for infratentorial and supratentorial subgroups. Positive Δ values indicate improvement in overall swallowing ability (MASA) and in self‐perceived swallowing‐related quality of life (SWAL‐QoL). The asterisk denotes a statistically significant difference identified in exploratory subgroup analyses.

Consistent findings emerged for the PAS and the MASA, both of which improved significantly over time in the active and sham arms (Figures 3 and 4).

Patient‐reported outcomes on the SWAL‐QoL also demonstrated a marked improvement at 6 weeks compared with baseline in both groups (Figure 4). As for the clinical scales, the degree of change did not differ significantly between anodal and sham stimulation.

### 3.3 Subgroup Exploratory Analyses

Exploratory analyses were performed to investigate potential moderators of treatment response, including lesion location, intervention timing, and sex (Tables S2 and S3).

When stratified by lesion site, patients with infratentorial strokes tended to show larger improvements under active stimulation. For the MASA, the between‐arm difference (active vs. sham) was significant at 2 weeks (*p* = 0.04) but not at 6 weeks (*p* = 0.08). For the DOSS, a trend was observed at 2 weeks (*p* = 0.07) but not at 6 weeks (*p* = 0.16), while PAS differences were not significant at either time point (*p* = 0.5 and *p* = 1). In supratentorial cases, no between‐arm differences were observed across outcomes (Figures 3 and 4).

Regarding intervention timing, no significant differences in swallowing improvement were found between patients treated early (≤ 4 weeks post‐onset) and those treated later (> 4 weeks), either overall or within the active and sham arms (Table S2).

Sex‐related differences were examined across the entire cohort. Males showed larger gains in MASA and SWAL‐QoL than females (Table S3).

### 3.4 Correlation Analyses

Spearman correlations between delta scores (DOSS, MASA, PAS, and SWAL‐QoL) and baseline measures highlighted that greater baseline dysphagia severity predicted larger gains. Lower baseline MASA was associated with greater improvements in MASA (Δ2 weeks *r* = −0.54, *p* = 0.002; Δ6 weeks *r* = −0.52, *p* = 0.004) and PAS (Δ2 weeks *r* = −0.54, *p* = 0.002). Higher baseline PAS correlated with larger gains in DOSS (Δ6 weeks *r* = −0.56, *p* = 0.002), PAS (Δ2 weeks *r* = −0.51, *p* = 0.004), MASA (Δ6 weeks *r* = −0.41, *p* = 0.03), and SWAL‐QoL (Δ6 weeks *r* = −0.46, *p* = 0.01). Among non‐swallowing covariates, higher baseline NIHSS correlated with greater improvement in PAS at 2 weeks (*r* = 0.39, *p* = 0.007); no other correlations reached significance.

## 4 Discussion

This randomized, double‐blind, sham‐controlled multicenter trial evaluated the effects of bilateral anodal tDCS combined with intensive speech‐language therapy in post‐stroke oropharyngeal dysphagia. Both active and sham groups showed significant and progressive improvement across all swallowing outcomes, highlighting the strong effect of structured and prolonged rehabilitation. No overall superiority of active stimulation was observed. Exploratory analyses suggested larger functional gains in patients with infratentorial lesions. Overall, these findings indicate that intensive therapy represents the main driver of recovery, while tDCS may confer additional benefit in selected subgroups. Among the outcome measures, the DOSS appeared to be the most sensitive in capturing sustained improvement over time.

Compared with previous studies, this trial enrolled patients in the subacute and early chronic stages (72 h–6 months post‐stroke), all of whom were clinically stable and actively engaged in rehabilitation. Improvements likely reflected a combination of therapy‐related effects and spontaneous recovery, whose relative contributions cannot be determined within this design. A no‐treatment control group was not included for ethical reasons. In contrast to prior findings suggesting enhanced responsiveness during early rehabilitation [13, 14, 16, 17], no significant differences in swallowing improvement were observed between early‐ and late‐treated patients, either overall or within treatment arms. These findings suggest that responsiveness to intensive therapy and neuromodulation remains relatively stable across the subacute–early chronic window. Although a contribution from spontaneous recovery cannot be excluded, the results support that structured rehabilitation can lead to meaningful improvement beyond the early post‐stroke phase, with a possible additional contribution of neuromodulation in selected subgroups.

The lack of a significant difference between active and sham stimulation may reflect a ceiling effect related to the high intensity and duration of the behavioral program. Participants received 6 weeks of structured 40‐min daily SLT, extending beyond the stimulation phase and exceeding the intensity of most previous tDCS trials for post‐stroke dysphagia. Earlier studies typically combined tDCS with shorter or less intensive therapy [14, 16, 18], and in acute settings rehabilitation was often limited by reduced cooperation or medical instability [16, 25]. The comprehensive SLT regimen likely maximized recovery in both groups, leaving limited room for additional neuromodulatory effects. This interpretation is consistent with recent meta‐analyses showing that when conventional therapy is highly intensive, incremental effects of tDCS are difficult to detect [26, 27]. In addition, the inclusion of patients with mild‐to‐moderate dysphagia may have further contributed to a ceiling effect, reducing the detectable margin for improvement and potentially limiting the ability to demonstrate an additional benefit of active stimulation over sham. Accordingly, the absence of group‐level differences likely reflects the dominant effect of intensive SLT rather than a lack of efficacy of tDCS.

Future studies may explore different combinations of SLT intensity and neuromodulation, as a tDCS‐only arm would raise ethical concerns given that SLT is the standard of care.

Stimulation montage and cortical targeting are critical for interpreting these results. The present protocol employed bilateral anodal stimulation over the primary swallowing cortices to engage both hemispheres. Bilateral stimulation has been proposed to enhance excitability across the distributed swallowing network and may produce broader effects than unilateral approaches [13, 17, 27, 28]. However, clinical efficacy likely depends on lesion location and recovery phase. In the early phase, contralesional anodal stimulation may enhance compensatory activation of the intact swallowing cortex [16], whereas at later stages bilateral or ipsilesional montages may strengthen residual cortical representations [10, 12, 27]. Given variability in lesion site and reorganization patterns, a bilateral montage may not ensure optimal cortical engagement in all patients. Modeling studies suggest that multi‐electrode montages can generate more focused fields and reach subcortical or brainstem structures with reduced dispersion [29]. Accordingly, future multicenter trials with stratified enrollment and imaging‐guided targeting are needed to define the most effective montage across recovery phases and lesion profiles.

Exploratory subgroup analyses raised the hypothesis that bilateral anodal stimulation may be particularly advantageous in patients with infratentorial stroke. In this subgroup, active stimulation was associated with greater functional gains than sham, reaching significance for MASA scores at 2 weeks and showing a trend for DOSS. However, these findings should be interpreted with caution, as the subgroup analyses were not powered for definitive inference and the number of infratentorial cases was limited. Therefore, these observations should be considered hypothesis‐generating and require confirmation in larger adequately powered stratified studies. Notwithstanding the limited number of infratentorial cases, this finding is noteworthy, as these lesions frequently cause severe dysphagia due to disruption of brainstem swallowing circuits. This population has been underrepresented in previous tDCS trials, resulting in limited evidence on responsiveness to neuromodulation. These results extend the findings of Mao et al. [18], who reported benefits of contralesional anodal tDCS after acute brainstem stroke, suggesting that bilateral stimulation may engage both hemispheric swallowing cortices to facilitate supratentorial compensation beyond the very acute phase. Given the bilateral corticobulbar control of swallowing [12, 30, 31], this mechanism appears physiologically plausible, although the relative advantage of bilateral over unilateral stimulation remains unclear. Future studies focusing on infratentorial stroke patients are needed to clarify optimal montage and timing. It also remains uncertain whether tDCS exerts direct effects on brainstem structures; while cortical and corticobulbar mechanisms are likely predominant, subcortical modulation cannot be excluded [28, 29].

Exploratory analyses provided additional insights into factors influencing swallowing recovery. Male patients showed larger improvements in MASA and SWAL‐QoL scores than females, likely reflecting greater baseline severity and a wider recovery margin rather than sex‐specific neuroplasticity differences. This interpretation is consistent with previous evidence identifying baseline dysphagia severity as a key determinant of treatment responsiveness, with more severely impaired patients often achieving proportionally greater gains [16, 17, 26, 27]. Correlation analyses further showed that greater baseline dysphagia severity (lower MASA and higher PAS scores) and higher baseline NIHSS were associated with larger improvements across multiple outcome measures. Infratentorial patients receiving active tDCS also demonstrated larger gains than those in the sham group, which may similarly reflect greater baseline impairment and a wider recovery margin, potentially further enhanced by tDCS effects in severe dysphagia. This hypothesis warrants further investigation to clarify the relative contribution of baseline severity and lesion location to treatment responsiveness.

Despite the strengths of the standardized multicenter design, several limitations should be acknowledged. The sample size limited the statistical power of subgroup analyses, particularly those stratified by lesion site. Moreover, the sample size calculation was based on expected within‐group changes in swallowing outcomes. Therefore, the study may have been underpowered to detect modest between‐group differences between active and sham stimulation.

In addition, the broad inclusion window (72 h–6 months) likely introduced heterogeneity in spontaneous recovery trajectories and time‐dependent neuroplasticity mechanisms. This variability may have reduced the ability to detect differential treatment effects between active and sham stimulation, particularly given the dynamic changes in swallowing recovery occurring across the subacute and early chronic phases after stroke. At the same time, including patients across this clinically relevant recovery window improves the generalizability of our findings to routine neurorehabilitation practice.

The bilateral montage, although physiologically sound, may have generated diffuse current fields with reduced focality compared with unilateral or high‐definition configurations. Electrode positioning based on standardized scalp coordinates rather than individualized neuronavigation or TMS mapping may have further contributed to interindividual variability, although this approach is consistent with previous tDCS trials in post‐stroke dysphagia [9, 16]. Future studies combining tDCS with functional or neurophysiological mapping may improve targeting and reproducibility.

In conclusion, this multicenter randomized trial demonstrates that, within the subacute–early chronic window, intensive and prolonged SLT leads to meaningful improvement in post‐stroke oropharyngeal dysphagia irrespective of stimulation condition. This finding highlights the importance of access to structured, long‐duration rehabilitation even in early chronic patients [32]. Exploratory findings suggest a possible additional benefit of bilateral tDCS in infratentorial lesions, which requires confirmation in larger, stratified studies. Given stroke heterogeneity and time‐dependent neural plasticity, future research should adopt adequately powered designs incorporating neuroimaging and neurophysiological markers to identify responders and optimize stimulation targeting. Overall, intensive behavioral rehabilitation remains the cornerstone of dysphagia recovery, while the contribution of tDCS likely depends on lesion characteristics and baseline severity.

## Author Contributions

**Simone Pierro:** investigation, writing – review and editing, data curation. **Simone Mauramati:** investigation, writing – review and editing. **Antonello Grippo:** investigation, writing – review and editing. **Valeria Pingue:** conceptualization, methodology, investigation, writing – review and editing. **Giulia Bertino:** investigation, writing – review and editing. **Emanuela Concas:** data curation, investigation, writing – review and editing. **Tommaso Bocci:** conceptualization, methodology, investigation, writing – review and editing. **Carla Giudice:** data curation, investigation, writing – review and editing. **Giuseppe Cosentino:** conceptualization, methodology, investigation, formal analysis, funding acquisition, writing – original draft. **Ilaria Pellegrini:** data curation, investigation, writing – review and editing. **Antonio Nardone:** supervision, writing – review and editing. **Alberto Priori:** writing – review and editing, supervision. **Nicole Pizzorni:** conceptualization, methodology, writing – review and editing. **Enrico Alfonsi:** conceptualization, supervision, writing – review and editing. **Valentina Grillo:** data curation, investigation, writing – review and editing. **Sara Rocca:** data curation, investigation, writing – review and editing. **Antonio Schindler:** supervision, writing – review and editing. **Shaheen Hamdy:** supervision, writing – review and editing. **Francesca Cecchi:** conceptualization, methodology, investigation, writing – review and editing. **Cristina Tassorelli:** supervision, writing – review and editing, funding acquisition.

## Funding

This study was supported by the Italian Ministry of Health (Ricerca Corrente 2025–2027).

## Conflicts of Interest

Shaheen Hamdy is Chief Scientific Officer at Phagenesis Ltd. and holds stocks and shares in the company. All other authors declare no competing financial interests or personal relationships that could have appeared to influence the work reported in this paper.

## Supporting information


**Table S1:** Structure and content of the swallowing rehabilitation program.
**Table S2:** Comparison of delta scores (change from baseline) in swallowing outcomes between patients treated early (≤ 4 weeks from stroke onset) and those treated later (> 4 weeks). Values are expressed as median (interquartile range). Analyses were conducted for the entire cohort (active and sham combined) and separately within each treatment arm (active and sham tDCS). Comparisons between early and late subgroups were performed using the Mann–Whitney *U* test.
**Table S3:** Comparison of delta scores (change from baseline) in swallowing outcomes between male and female patients. Values are expressed as median (interquartile range). Analyses were conducted on the entire sample (active and sham combined). Comparisons were performed using the Mann–Whitney *U* test.

## Data Availability

The anonymized dataset supporting the findings of this study is available on Zenodo at https://doi.org/10.5281/zenodo.17543207.
