# Supplementary material for: Intensive Rehabilitation With Adjunctive Bilateral Anodal tDCS in Post‐Stroke Dysphagia: A Multicenter Randomized Controlled Trial
Source: Eur J Neurol. 2026 Jul 1;33(7):e70686. doi: 10.1111/ene.70686 (PMC13321463; doi:10.1111/ene.70686)
Supplement: Supplementary file 1 — Table S1: Structure and content of the swallowing rehabilitation program. Table S2: Comparison of delta scores (change from baseline) in swallowing outcomes between patients treated early (≤ 4 weeks from stroke onset) and those treated later (> 4 weeks). Values are expressed as median (interquartile range). Analyses were conducted for the entire cohort (active and sham combined) and separately within each treatment arm (active and sham tDCS). Comparisons between early and late subgroups were performed using the Mann–Whitney U test. Table S3: Comparison of delta scores (change from baseline) in swallowing outcomes between male and female patients. Values are expressed as median (interquartile range). Analyses were conducted on the entire sample (active and sham combined). Comparisons were performed using the Mann–Whitney U test. [file ENE-33-e70686-s001.docx]

**Supplementary Materials**

| Phase | Duration | Session frequency | Treatment | Session structure | Exercises and techniques | Therapeutic goals |
| --- | --- | --- | --- | --- | --- | --- |
| Week 1–2 | 2 weeks (10 sessions) | 5 days/week, 40 min/day | SLT started during tDCS and continued immediately after | **First 20 min:** concurrent tDCS and SLT  **Following 20 min:** SLT continued without stimulation | Effortful swallow, Mendelsohn, Masako, super-supraglottic swallow, Shaker head lift, Chin-tuck against resistance,  tongue-strengthening, orofacial and laryngeal mobility exercises, postural adjustments, and compensatory strategies | Facilitate cortical-bulbar plasticity; enhance safety and efficiency of swallowing |
| Week 3–6 | 4 weeks (20 sessions) | 5 days/week, 40 min/day | SLT only (no stimulation) | SLT alone: 40 min | Continuation and progression of individualized swallowing and non-swallowing exercises, postural and compensatory strategies | Consolidate functional gains; improve endurance and volitional control |

**Supplementary Table 1.** Structure and content of the swallowing rehabilitation program.

SLT: speech and language therapy; tDCS: transcranial direct current stimulation.

|  | All patients | | | Active (anodal) tDCS | | | Sham tDCS | | |
| --- | --- | --- | --- | --- | --- | --- | --- | --- | --- |
|  | Early | Late | p | Early | Late | p | Early | Late | p |
| ∆ DOSS (2 weeks – baseline) | 1.0 (2.0) | 1.0 (1.0) | 0.89 | 1.0 (2.0) | 1.0 (2.0) | 0.24 | 0.0 (2.0) | 1.0 (1.0) | 0.97 |
| ∆ DOSS (6 weeks – baseline) | 1.0 (2.0) | 1.0 (2.0) | 0.78 | 1.0 (2.0) | 2.0 (4.0) | 0.17 | 1.0 (2.0) | 1.0 (2.0) | 0.62 |
| ∆ PAS (2 weeks – baseline) | 0.0 (0.0) | 0.0 (1.0) | 0.47 | 0.0 (0.0) | 0.0 (0.0) | 0.81 | 0.0 (0.0) | 0.0 (1.0) | 0.34 |
| ∆ PAS (6 weeks – baseline) | 0.0 (2.0) | -1.0 (2.0) | 0.13 | 0.0 (2.0) | -1.0 (2.0) | 0.17 | 0.0 (1.0) | -1.0 (2.0) | 0.50 |
| ∆ MASA (2 weeks – baseline) | 7.0 (15.0) | 9.0 (22.0) | 0.67 | 7.0 (10.0) | 15.0 (26.0) | 0.65 | 0.0 (24.0) | 6.0 (14.0) | 0.52 |
| ∆ MASA (6 weeks – baseline) | 11.0 (18.0) | 10.0 (20.0) | 1.00 | 11.0 (17.0) | 8.0 (55.0) | 0.88 | 7.0 (22.0) | 11.5 (18.0) | 0.92 |
| ∆ Swal-QoL (6 weeks – baseline) | 44.5 (170.0) | 20.0 (80.0) | 0.43 | 107.0 (163.0) | 31.0 (145.0) | 0.65 | 30.0 (174.0) | 18.0 (33.0) | 0.94 |

**Supplementary Table 2**. Comparison of delta scores (change from baseline) in swallowing outcomes between patients treated early (≤ 4 weeks from stroke onset) and those treated later (> 4 weeks). Values are expressed as median (interquartile range). Analyses were conducted for the entire cohort (active and sham combined) and separately within each treatment arm (active and sham tDCS). Comparisons between early and late subgroups were performed using the Mann–Whitney U test.

DOSS: Dysphagia Outcome and Severity Scale; PAS: Penetration–Aspiration Scale; MASA: Mann Assessment of Swallowing Ability; SWAL-QoL: Swallowing Quality of Life questionnaire.

|  | Female | Male | p |
| --- | --- | --- | --- |
| ∆ DOSS (2 weeks – baseline) | 1.0 (1.0) | 1.0 (1.0) | 0 .18 |
| ∆ DOSS (6 weeks – baseline) | 1.5 (1.0) | 1.0 (2.0) | 0 .28 |
| ∆ PAS (2 weeks – baseline) | 0.0 (0.0) | 0.0 (1.0) | 0 .19 |
| ∆ PAS (6 weeks – baseline) | -0.5 (2.0) | -0.5 (2.0) | 0 .99 |
| ∆ MASA (2 weeks – baseline) | 18.5 (20.0) | 4.5 (11.5) | **0.004** |
| ∆ MASA (6 weeks – baseline) | 20.5 (23.0) | 7.0 (18.0) | **0.01** |
| ∆ Swal-QoL (6 weeks – baseline) | 157.0 (146.0) | 14.5 (60.0) | **0.0003** |

**Supplementary Table 3.** Comparison of delta scores (change from baseline) in swallowing outcomes between male and female patients. Values are expressed as median (interquartile range). Analyses were conducted on the entire sample (active and sham combined). Comparisons were performed using the Mann–Whitney U test.

DOSS: Dysphagia Outcome and Severity Scale; PAS: Penetration–Aspiration Scale; MASA: Mann Assessment of Swallowing Ability; SWAL-QoL: Swallowing Quality of Life questionnaire. Bold p-values indicate statistically significant differences (p < 0.05).
